# Realising health justice in Palestine: beyond humanitarian voices

**DOI:** 10.1186/s13031-024-00634-0

**Published:** 2025-02-05

**Authors:** James Smith, Sara el-Solh, Layth Hanbali, Sawsan Abdulrahim, Sali Hafez, Samer Abuzerr, Bram Wispelwey, Mads Gilbert, Mohammed Seyam, Bassam Abu Hamad, Dorotea Gucciardo, Fahd Haddad, Rasha Khoury, Amira Nimerawi, David Mills, Ghassan Abu-Sittah

**Affiliations:** 1 Centre for Humanitarianism and Social Inclusion, Institute of Epidemiology and Health Care, University College London, London, UK; 2 Medact, London, UK; 3 Institute of Community and Public Health, Birzeit University, Birzeit, Palestine; 4 American University of Beirut, Beirut, Lebanon; 5 Palestine Program for Health and Human Rights, François-Xavier Bagnoud Center for Health and Human Rights, Harvard University, Boston, USA; 6 Health in Humanitarian Crises Centre, London School of Hygiene and Tropical Medicine, London, UK; 7 Department of Medical Sciences, University College of Science and Technology, Khan Younis, Gaza, Palestine; 8 University Hospital of North-Norway, Tromsoe, Norway; 9 Faculty of Medicine, Al-Quds University, Gaza, Palestine; 10 Global Business School for Health, Faculty of Population Health Sciences, University College London, London, UK; 11 School of Public Health, Al-Quds University, Gaza, Palestine; 12 The Glia Project, Gaza, Palestine; 13 Starling Centre for Just Technologies & Just Societies, Western University, London, Canada; 14 Department of Emergency Medicine, Deir Al Balah, Shohada Al Aqsa Hospital, Gaza, Palestine; 15 Boston University School of Medicine, Boston, USA; 16 Health Workers for Palestine, London, UK; 17 Conflict Medicine Program, American University of Beirut, Beirut, Lebanon

**Keywords:** Gaza, Palestine, Israel, Genocide, Occupation, Settler colonialism, Humanitarianism, Determinants of health

## Abstract

In the shadow of Israel’s ongoing genocide throughout occupied Palestine, this article examines the moral, political, and epistemic responsibilities of humanitarian, public health, global health, medical and related communities of practice amid profound violence and injustice. In this paper we offer a three-part critique of a May 2024 commentary by Karl Blanchet and colleagues, ‘Rebuilding the health sector in Gaza: Alternative humanitarian voices’ (Blanchet et al. in Confl Heal 18:42, 2024). We argue that in relation to the post-genocide reconstruction of the health system in Gaza, the authors have failed to adequately contextualise their analysis, or the health system reconstruction policy proposal as commissioned by the Gaza Health Initiative [Gaza Health Rebuilding Initiative (GHRI), Future of the Gaza health system: Needs assessment. https://static1.squarespace.com/static/656c4ff5b219116063f8099e/t/65c30c4d3e80fd21f1165b57/1707281499127/Final+first+draft+-+Gaza+health+system+report.pdf. Accessed 8 November 2024], and as such have failed to elucidate the root causes of what manifests most immediately as an entirely manufactured humanitarian crisis. This in turn undermines the substance and utility of the recovery proposal as it has been presented. We also argue that the authors have failed to afford due recognition to several key health actors in Palestine, in particular the Palestinian Ministry of Health in Gaza and UNRWA, and that this subtle but critical act of erasure further undermines the relevance and applicability of their recovery proposal as presented. In response we emphasise the centrality of Palestinian perspectives, and the need to uphold Palestinian leadership in all stages of the recovery process. Thirdly, we take issue with the suggested chronology and substance of the recovery proposal as presented, which overlooks response and recovery efforts already underway and led by Palestinians in Gaza, and which overstates ultimately harmful intervention strategies such as networks of internationally managed field hospitals. Against the backdrop of a very limited window for what we consider morally justifiable humanitarian engagement in Gaza, we join many others who have called for a paradigm shift away from the inertia of supposed objectivity and claimed neutrality that function to perpetuate injustice. Instead, we call for the collective practice of critical advocacy, solidarity with people affected by injustice and oppression, and an emancipatory politics in pursuit of justice in Palestine.

In a May 2024 commentary [1], Karl Blanchet and colleagues drew attention to ‘healthcare challenges caused by the recent war in Gaza’, though without mentioning Israel’s systematic violence amounting to genocide and ethnic cleansing against the Palestinian people [3–6]. The authors do not name Israel except for cursory reference to destruction caused by the Israeli military in one of its earlier, deadly escalations in Gaza in 2014. The authors shape their paper around the ‘First International Conference to Rebuild the Health Sector in Gaza’, held 7–8 February 2024 in Amman [7], before offering their perspective on steps required to rebuild Gaza’s health sector including circumventing an environment of ‘donor fatigue’ [1].

Blanchet et al.’s choice of an almost exclusively humanitarian frame and passive language in their paper [1], which also sketches out the contours of a multi-phase recovery plan for Gaza commissioned by the Gaza Health Initiative (GHI) [2], demands critique and rebuttal. This is particularly urgent for several reasons. Firstly, Israel’s acute-on-protracted [8] genocidal violence against the Palestinian people continues unabated. Failing to explicitly acknowledge this reality and its underlying settler colonial root causes [9–11], while simultaneously proposing a series of subsequent actions, risks obscuring and ultimately derailing genuine response and recovery efforts, and tacitly accepting reversion to the violent status quo of Israeli occupation and apartheid, marked by repeated cycles of destruction and reconstruction [12]. Secondly, calls for recovery within the structure of the hegemonic humanitarian system, with its fleeting programmatic engagement, heavily conditional donor support, pseudo-apolitical positioning, and narrow interpretations of “crisis”, will clearly cause more harm than benefit in Gaza—and throughout occupied Palestine—in the absence of bold, comprehensive proposals that transcend the limited paradigmatic imagination of mainstream humanitarianism. Here we observe that while Blanchet et al. seek to promote ‘alternative humanitarian voices’ [1], the substance of their analysis and proposal as summarised fall squarely within the boundaries of conventional humanitarian policy formulation and response, which are largely unfit for purpose in Palestine or any other context. The critique of the harms caused by – and violence enacted through—humanitarian interventions in Palestine is well established, and recognises the wider implications of humanitarianised narratives that obscure the legal basis for, and political imperative of, the Palestinian struggle for liberation [13–15]. As anthropologist Sa’ed Atshan has argued, ‘The tragedy of Gaza is fundamentally a moral crisis of a global system entrenching Israeli impunity where humanitarianism has become a substitute for politics and accountability’ [16].

## Naming Israel’s genocidal violence

In a May 2024 interview, historian Rashid Khalidi highlighted a notable ‘discursive shift’ in public engagement on Israel: ‘today we are talking about apartheid, genocide, settler colonialism, and accountability’ [17]. In the same interview Khalidi goes on to describe attempts by ‘Western politicians and media’ to reverse this shift, the success of which would reinstate an ‘unjust status quo’. Put concisely by UN Special Rapporteur Francesca Albanese, ‘it is important to call a genocide a genocide’ [18]. Both Khalidi and Albanese’s insights underscore the critical importance of language [19], framing, and analytical depth in shaping collective mobilisation and response strategies in and for Gaza today, as well as the form of meaningful engagement over the long term.

Blanchet et al. fail to name the full extent of Israel’s genocidal violence against the Palestinian people, preferring instead to use the vague and misleading phrase ‘war in Gaza’ [1]. On the day of the Amman Conference, the Palestinian Ministry of Health in Gaza (hereafter, the Ministry of Health) reported that at least 27,708 Palestinians had been killed, and a further 67,147 wounded, by sustained Israeli ground, air, and naval attacks throughout Gaza [20]. Among a litany of evolving war crimes, on the same day the Palestine Red Crescent Society (PRCS) confirmed that PRCS paramedic Mohammed Al-Omari had been killed and another two people injured when Israeli occupation forces fired directly on them as they transferred patients from Al Ahli Hospital in Gaza City [21]. In Khan Younis, Israeli occupation forces laying siege to Al Amal Hospital shot and wounded a patient and a caretaker who were within the hospital premises [22].

Several descriptive elements in Blanchet et al.’s paper misrepresent and downplay the severity of Israel’s violence and the longstanding injustices perpetrated against the Palestinian people. For instance, the authors state that at the time of writing (31st March 2024) ‘the border remains closed’ [1]. Rather, Israel has exerted even greater control over Gaza since imposing a comprehensive blockade and siege in 2007 against the backdrop of its protracted occupation, periodically blocking the movement of people and essential items through a limited number of heavily militarised entry points that cross the armistice line. Since October 2023, Israel has intensified its control by intentionally limiting the entry of aid and commercial trucks, banning essential items such as insulin pens, orthopedic instruments, crutches, and anaesthetic medications [23], hindering the movement of people into and out of Gaza (including people in need of immediate medical and surgical care), and deliberately obstructing access to water, food, electricity, and fuel. All of these actions are in breach of Israel’s minimum obligations as an occupying power [24]. This system of complete control, with influence also exerted over neighbouring Egypt and Palestine’s maritime and air spaces, allows Israel to effectively titrate the means of life and death for Palestinians living in Gaza [25]. This is not simply a matter of border closure, but rather systematic occupation that creates what philosopher Achille Mbembe describes as a ‘death-world’ in which Palestinians living in Gaza are forced to live in a prolonged state of living death [26].

Elsewhere Blanchet et al. rightly emphasise that the ‘safety and security of health professionals need to be ensured’ [1], but do not make reference to the fact that the systematic dismantling of the health sector, including the targeting of Palestinian healthcare workers—and Palestinian people more broadly—forms a central pillar of Israel’s military strategy [27]. In Gaza, this includes Israel killing more than 1000 Palestinians working in the health sector [28, 29]; repeated Israeli raids and attacks on health facilities, ambulances, and other healthcare infrastructure [30–33]; the detention, torture, and killing of Palestinians – including healthcare workers—in Israeli detention camps; [28, 34] the explicit endorsement of attacks on healthcare facilities by Israeli doctors [35] ; and the involvement of Israeli health workers in acts of torture [36].

Blanchet et al.’s vague calls and perfunctory contextualisation are not only descriptively inadequate but also shape the discursive form and relative attention afforded to proposed responses in the short, medium, and long term. For instance, Blanchet et al. emphasise the importance of meeting basic needs, including that ‘clean water sources need to be restored’ [1], but do not mention that a substantial proportion of water in Gaza was already unfit for human consumption due to exceptionally high levels of contamination and salinity prior to October 2023 [37]. Furthermore, they omit the critical fact that Israel exerts complete control over Gaza's water supply: a cornerstone of its ongoing occupation of Palestine [38]. What does restoration look like under such circumstances? By sidestepping the root causes of precarious access to safe water in Gaza, incremental improvements in sanitation and water quality become the yardstick for a successful public health response, rather than efforts to bring about an end to the occupation.

While recognising the urgent need for sustained access to safe water as a fundamental human right, the recovery phases as presented by Blanchet et al. do not substantively address the underlying systems of settler colonialism, apartheid, and occupation that precipitate life-altering and life-ending water availability and water quality issues. These systems systematically deprive Palestinians of their most basic physiological needs, including water and food. They are imposed in order to subjugate, dispossess, and ethnically cleanse Palestinians by making their spaces unlivable, as has been openly and repeatedly stated by Israeli officials [39]. Despite the centrality of settler colonialism as a foundational determinant of health [40]**, **Blanchet et al. make only cursory reference to the need to account for the critically important ‘political, social, economic, and environmental determinants of health’ [1]. The authors do not attempt to name those determinants, nor acknowledge that the political aim of suffocating and dispossessing Palestinians requires a series of fundamentally political solutions. As such the authors fail to confront the entrenched injustices perpetrated against the Palestinian people.

Centres and think tanks dedicated to research and policy formulation in the fields of humanitarian public health and humanitarian medicine wield significant—and as yet, largely understudied—epistemic power in the framing of, and wider systemic engagement with, the gravest forms of violence and injustice worldwide [41]. This influence carries with it a profound moral and epistemic responsibility to uphold a commitment to descriptive accuracy and depth, and value-driven analytical rigor. Recent controversies surrounding the ways in which an influential academic humanitarian health centre in London selectively described and failed to contextualise the current violence in Gaza (while others have opted for similarly harmful misrepresentations, complete silence, or proactive silencing) demonstrate that such centres and their host institutions are also implicated in wider processes of epistemic coloniality [42, 43].

## Voices central to Gaza’s health system recovery

It is striking that Blanchet et al. acknowledge the significance of diverse voices in their paper, yet omit specific Palestinian contributions and only refer by name to large international organisations alongside undefined ‘local stakeholders’ in relation to actors that should be involved in their proposed recovery efforts [1]. Relatedly, notably absent from their roll call of conference participants is mention of Palestinian health professionals, the Ministry of Health, or UNRWA representatives, despite their active participation in multiple panels during the February 2024 Amman Conference. Choosing not to explicitly acknowledge these actors aligns with a concerted effort on the part of Israel to vilify Palestinians and Palestinian and other organisations working in service of the Palestinian people. As such, these omissions represent subtle but critical acts of erasure by failing to afford due recognition to the pivotal role played by local and national actors that are central to health service delivery in Gaza, whose experience, knowledge, and leadership has been indispensable, and will remain critical in the coming period.

Any sustainable future for the Palestinian healthcare system hinges crucially on the involvement and strategic leadership of the Ministry of Health. The Ministry has played a pivotal role in safeguarding health in Gaza throughout decades of Israel’s protracted occupation, and now the genocide. Despite the Israeli military’s relentless attacks on healthcare and allied workers, and the widespread destruction of health infrastructure, the Ministry has continued to coordinate and oversee health service delivery, medical logistics, the cooperation of international organisations, health workforce planning, maintenance of health information systems, and reporting on the impact of the occupation - and now the genocide - on the Palestinian people and the health system.

UNRWA has also played an integral role in the provision of health, education, social and other essential services to the Palestinian people in the wake of the 1947–49 Nakba, during which time approximately 750,000 Palestinians – and nationals of another two dozen countries—were violently ethnically cleansed from Palestine by Zionist terror groups (and later the Israeli military) [44, 45]. The creation of UNRWA was mandated by UN Resolution 302 (IV) in 1949 [46], which in turn was shaped by UN Resolution 194 (III) [47] that explicitly affirms the right of return and reparations for Palestine refugees. While the creation of UNRWA was necessitated in part by a failure to create a just mechanism for the rightful return of Palestine refugees to their homes in the aftermath of the Nakba, coordinated efforts over recent years to defund and undermine UNRWA by Israel, the USA and a multitude of other “Global North” governments, are an attempt to further weaken the political commitment to the UN-mandated Palestinian right of return, and to further marginalise Palestine refugees [45, 48, 49]. Such actions constitute collective punishment through the denial of UNRWA's vital health, educational, social and other services to Palestinians throughout occupied Palestine and to Palestine refugees in neighbouring countries [50]. In the context of ongoing attempts to undermine and dismantle UNRWA, it is incumbent upon individuals speculating over the future of health and healthcare systems in Gaza to explicitly recognise the critical role of UNRWA as a key actor in response and recovery efforts. This includes working to reverse the pressures put on UNRWA over several years, which have led it to reduce services and crack down on staff and service recipients’ rights.

Finally, Blanchet et al.'s paper alludes to the predominance of external perspectives in current conversations concerning the present and future of health system response and recovery in Gaza. While the authors acknowledge the inclusion of Palestinian members in a new international health coalition for Gaza, they also refer to individuals from various other countries who ‘have been working in Gaza for over twenty years’, and to coalition ‘skills and dedication available to help Gazans’ [1]. Not only does this language bely that much of the coalition is situated outside Gaza and wider Palestine, but it also implies the deprivation of Palestinian agency, a logic of external charity, and a process that has centred a foreign gaze and pose [51]. We take this opportunity to underscore the centrality of Palestinian expertise and perspectives in the formulation and implementation of all stages of the response and recovery process. Any proposals that undermine or overlook Palestinian leadership, that fail to integrate the breadth of Palestinian skills and knowledge, or that deny Palestinian ownership, must be unequivocally rejected [52].

## On the substance of recovery proposals

In addition to concerns regarding the depth and accuracy of contextualisation, and the voices and perspectives central to recovery efforts, it is also necessary to challenge the substance of the recovery proposal as presented in Blanchet et al.’s paper [1]. This concerns issues with the stepwise chronology of proposed actions, and material strategies that are likely to have a major negative impact on health system recovery.

### Rebuilding is already underway with Palestinian leadership

Efforts to rebuild and refurbish damaged hospitals as outlined in phase three—the ‘long-term reconstruction phase’ -[1] of the recovery proposal as described by Blanchet et al. are already well underway. These remarkable efforts have been driven forward with Palestinian leadership and support from Palestinian healthcare workers and community volunteers, along with coordination from several health actors such as the Ministry of Health and PRCS, and with some assistance from the WHO and national and international non-governmental organisations (INGOs). On the day of his release after surviving more than seven months in Israeli captivity, Dr Muhammad Abu Salmiya, a paediatrician and the director of Al Shifa Hospital, announced, ‘I promise you and the world that we will rebuild [Shifa] medical complex’ [53]. Despite extensive damage by the Israeli military to almost all health facilities in Gaza, and the discovery of mass graves at several hospital sites [54], at the time of writing parts of Al Shifa, Nasser, Al Amal, Indonesian, and other hospitals and clinics have been refurbished and partially reopened, allowing healthcare workers to once again provide emergency, critical care, maternity, dialysis, surgical and other essential services in Khan Younis, Gaza City, and North Gaza [55–58].

### Field hospitals undermine the pre-existing Palestinian health system

Relatedly, the expansion of field hospitals throughout Gaza raises a multitude of ethical and political concerns and should not be promoted as part of any recovery proposal without several important caveats.

Notably, the establishment of field hospitals in Gaza since October 2023 did not form part of the initial Ministry of Health or WHO response strategies. Such facilities have been reluctantly incorporated as pragmatic measures in the face of the Israeli military’s unprecedented and systematic destruction of the pre-existing healthcare system. The Ministry of Health and WHO initially favoured international support within existing health facilities to avoid the creation of parallel systems, and to afford those facilities some limited additional protection through the visible presence of international organisations and their staff. It is incumbent upon anyone promoting a field hospital-based approach to clearly and roundly name and condemn the Israeli violence that precipitated the need for such temporary surge structures in the first instance, as MSF OCB did publicly when announcing the establishment of its field hospital in Zawayda [59].

Field hospitals have a very limited role to play when immediate population health needs far outweigh the response capacity of the health system. At the time of writing, several field hospitals are operational in the south and middle areas of Gaza and are receiving thousands of patients a day. These are admirable efforts, but must be time-bound, with a view to re-creating capacity within the pre-existing health system at the nearest possible opportunity. The obligation to safeguard Palestinian ownership, and to avoid the creation of parallel systems, requires that field hospitals managed by international organisations do not further undermine wider health system capacity with self-interested recruitment and remuneration practices and paternalistic response strategies, while adhering to the Ministry of Health’s quality standards, other cooperation requirements, and overarching authority. Instead, the current network of field hospitals almost entirely side-steps meaningful partnership with the Ministry of Health. Israel’s public endorsement and promotion of field hospitals managed by INGOs [60], while simultaneously continuing to attack Ministry of Health and PRCS facilities, forms part of a broader strategy intended to weaken and undermine the Ministry of Health and infrastructures for Palestinian self-determination by introducing externally administered – and easier to manipulate and control—networks of humanitarian government [61].

Additional moral demands clearly accrue when caring for people subjected to genocide and ethnic cleansing, with silence constituting a particularly egregious form of complicity. To date, several of the INGOs managing these field hospitals – like many other non-Palestinian humanitarian organisations currently working in Gaza—have failed to adequately advocate for their patients by refusing to clearly and consistently name Israeli violence—the direct and indirect effects of which they witness daily—or join the most basic calls for a sustained ceasefire [62, 63]. This state of complicity is further reinforced by statements made by the Israeli government, in which their involvement in humanitarian activities is overstated with claims that they have ‘facilitated and coordinated’ the establishment of the field hospitals [64]. Such inflated and unrefuted claims have formed a central pillar of Israel’s defense against accusations of genocide at the ICJ [65], and without clear and consistent repudiation further implicate humanitarian organisations in the most repugnant display of humanitarian complicity.

### Our collective responsibilities amid the ongoing genocide

While forward-thinking proposals for the rebuilding and recovery of the health system in Gaza are critically important, it is imperative that such proposals are grounded in the present moment. This requires full acknowledgement of the horrific scale and scope of ongoing genocidal violence perpetrated by Israel against the Palestinian people, from which naturally must follow urgent calls for an immediate end to all forms of Israeli aggression. This is a clear prerequisite to the full and sustained realisation of any recovery proposal. With this in mind it is striking that the most basic call for a ceasefire is only given cursory mention in the penultimate paragraph of Blanchet et al.’s paper [1].

International governments and multinational institutions certainly bear a moral, political and reparative obligation to contribute materially and financially to ongoing reconstruction efforts in Gaza. These obligations are particularly pronounced for governments that have actively participated or are otherwise complicit in the ongoing genocide, be it through the supply of military equipment or outright political and diplomatic indifference and neglect [66]. However, the urgency of these reconstruction efforts must not overshadow the overdue political pursuit of accountability and justice in Palestine, which must progress in tandem. Rebuilding and recovery efforts concern more than physical structures and demand a comprehensive reckoning with the underlying injustices that perpetuate states of protracted - or permanent – crisis and continual violence. Here we refer directly to Israel’s ongoing occupation of Palestine and the apartheid conditions progressively imposed on the Indigenous population of Palestine for more than 100 years.

Academic centres for the study of humanitarian public health often adopt a position of political neutrality akin to that of operational humanitarian organisations. While highly contentious and the subject of growing critique on both accounts [67–69], the latter attempt to justify neutrality based on its potential operational value as a means to secure access and minimise the risk posed to staff in situations of extreme violence. Research centres for the study of – and more commonly, study *for*—humanitarian action that remain concentrated in Geneva, Baltimore, London, Manchester, and a small number of other locations, do not bear those same risks. As such, academic neutrality is morally, intellectually and disciplinarily incoherent, particularly for those working in the fields of public health, medicine and nursing whose disciplinary and vocational values shape an additional moral imperative to act. Furthermore, many powerful academic centres engage not only in the passive process of remaining silent, but also in active processes of silencing and erasure, shaped in part by the influence of university donors and presumably in an attempt to remain palatable to future research funders [70–72]. These active processes expose the fallacy of a self-stated academic neutrality, and are demonstrative of the role played by many neoliberal academic institutions in the consolidation of epistemic and other forms of violence and oppression.

As scholar Rabab Ibrahim Abdulhadi explains, ‘what ‘cautious’ academics present as genuine claims for ‘neutrality’, and ‘lack of bias’, and respect for the privacy of ‘colleagues’ views’, only reifies the status quo, disciplines thought, and curtails critical thinking’ [73]. Structures of genocide, colonialism, and imperialism are all-consuming, and physically, materially and ideologically define relations between people, groups, nations, land and resources. They should therefore define how we understand and engage in all aspects of our moral and intellectual lives. With this in mind, it is incumbent upon academics, practitioners and policy-makers writing and engaging in all forms of public discourse to display moral and political courage in the form of clear statements of solidarity, along with actions that are experienced as such by all oppressed peoples, accompanied by unwavering demands for justice and disruptive action to that end.

## Imagining and enacting health futures in Palestine

We close by reflecting further on the profound moral, political, and epistemic responsibilities of humanitarian, public health, global health, medical and related communities of practice amid Israel’s ongoing genocide throughout occupied Palestine, and in Gaza in particular. This is not an academic exercise but a pressing call to action with profound implications. We implore our colleagues embedded within these disciplines to earnestly contemplate their roles and responsibilities in shaping a just and equitable future in Palestine, and in all places where violence and injustice have taken root.

Defending supposed neutrality in the face of genocide raises profound ethical questions, particularly for health workers, health policy advocates, and humanitarian researchers. How do we spend our time if not committed to value-driven indignation and engaged advocacy? What motivates us if not a desire for justice and liberation for all oppressed people? Who does neutrality serve, and who does it harm? By acknowledging that neutrality causes harm, on what basis do supposed health and humanitarian advocates insist on remaining neutral?

Naming sites of power and sources of injustice, and organising to expose and resist them, is central to what Nancy Krieger refers to as passionate epistemology and critical advocacy [74]. Public health has liberatory potential, rooted in, and shaped by, a desire for social justice [75]. This potential has been eroded by neoliberalism and the neoliberal university, with cadres of ‘value free’ experts now shaping the formulation and practice of public health [74, 76]. Public health, and its multiple articulations as global health, humanitarian public health, and the like, must belatedly centre a foundational commitment to bold advocacy and principled action in solidarity with people subjected to violence, oppression, and injustice. If certain academic disciplines and associated cadres of practitioners cannot muster the moral and political courage to confront genocide as the most horrific manifestation of orchestrated violence and systemic injustice, then we must urgently re-examine their moral, epistemic, and broader societal utility.

The future of health in Palestine hinges not only on rebuilding infrastructure and delivering services but on dismantling systems of oppression and advocating for the rights and dignity of all Palestinians. This work is solidarity-driven and shaped by a steadfast commitment to justice and accountability. Let us not falter in our duty to envision and enact a future where health and justice prevail.

## Data Availability

No datasets were generated or analysed during the current study.
